# Exposure–Response Relationship of Toxic Metal(loid)s in Mammals: Their Bioinorganic Chemistry in Blood Is an Intrinsic Component of the Selectivity Filters That Mediate Organ Availability

**DOI:** 10.3390/toxics13080636

**Published:** 2025-07-29

**Authors:** Manon Fanny Degorge, Jürgen Gailer

**Affiliations:** Department of Chemistry, University of Calgary, 2500 University Drive NW, Calgary, AB T2N 1N4, Canada

**Keywords:** biotransformation, bloodstream, plasma, red blood cells, plasma proteins, complex formation, mechanism of organ uptake

## Abstract

The gastrointestinal tract mediates the absorption of nutrients from the diet, which is increasingly contaminated with toxic metal(loid) species (TMs) and thus threatens food safety. Evidence in support of the influx of TMs into the bloodstream of the general and vulnerable populations (babies, children, pregnant women, and industrial workers) has been obtained by accurately quantifying their blood concentrations. The interpretation of these TM blood concentrations, however, is problematic, as we cannot distinguish between those that are tolerable from those that may cause the onset of environmental diseases. Since TMs that have invaded the bloodstream may perturb biochemical processes therein that will eventually cause organ damage it is crucial to better understand their bioinorganic chemistry as these processes collectively determine their organ availability. Thus, bioinorganic processes of TMs in the bloodstream represent selectivity filters which protect organs from their influx and ultimately determine the corresponding exposure-response relationships. The need to better understand selectivity filters prompted us to mechanistically disentangle them into the major bioinorganic chemistry processes. It is argued that the detoxification of TMs in the bloodstream and the biomolecular mechanisms, which mediate their uptake into target organs, represent critical knowledge gaps to revise regulatory frameworks to reduce the disease burden.

## 1. Introduction

The chronic low level exposure of human populations to toxic metal(loid) species (TMs), including As^III^, Cd^2+^, Hg^2+^ and CH_3_Hg^+^ and Pb^2+^ is becoming increasingly recognized as a global public health issue [1,2,3]. There are three main reasons why this problem has become of focal interest. The first is the realization that over the last decades it has been conclusively shown that progressively smaller exposure doses of TMs (10–50 μg/day) result in measurable adverse health effects in humans, including decreased cognitive abilities and neuropsychological development in children [4,5,6,7] with several TMs being established human carcinogens. Another reason is the environmental recalcitrance of these inorganic pollutants in the environment, which can explain why the implementation of measures to protect humans from toxic metal(loid)s emitted from local point sources in the past has not prevented the insidious regional contamination of agricultural soils with TMs in certain parts of the world [3]. An inevitable outcome of this undesirable development is the recent demonstration that the As, Cd, Hg, and Pb concentrations in some baby foods exceed their maximum permissible concentrations for bottled water mandated by the FDA 91-fold, 177-fold, 69-fold, and 5-fold, respectively [8]. While these data do not imply that all of these TMs are entirely absorbed into the bloodstream, they nevertheless exemplify that the most vulnerable members of our society are simultaneously exposed to several inorganic pollutants. The third reason is the comparatively recent realization that only a relatively small proportion of chronic diseases can actually be attributed to genetic factors alone [9], indirectly implying that environmental factors are likely to play an important role in disease etiology [4]. This realization also provides a plausible explanation for the considerable time delay between the start of a TMs exposure and the onset of overt adverse health effects [10].

While human exposure to TMs represent only one class of pollutants which contribute to the estimated ~9 million people that are killed by man-made pollutants every year [11], the latter number is most likely an underestimation and more work is needed to establish the true magnitude of the adverse effect that environmental factors exert on human health on a global scale. In this context, one is reminded of the work by Molina and Rowland who unraveled the chemical mechanisms which causally link the anthropogenic emission of CFCs into the atmosphere with the destruction of the stratospheric ozone hole thus causing adverse health effects in mammals [12]. Based on their discovery, a global reduction in the industrial production of CFCs eventually led to the Montreal Protocol, which was signed by 197 nations in 1987 and has since resulted in a significant shrinkage of the ozone hole over the Antarctic. In analogy to solving this manmade environmental chemistry-problem, a better understanding of the biomolecular mechanisms that causally link human TMs exposure with adverse organ-based health effects is urgently needed, as this represents the first step to considerably reduce the prevalence of preventable environmental diseases [4]. In principle, one needs to distinguish non-threshold effects which predominantly involve genotoxicity-based mechanisms from threshold effects which primarily involve biochemical processes and are the primary focus of this manuscript [13]. Given the difficulty of unraveling the latter biomolecular mechanisms that unfold in the GI-tract—bloodstream—organ system and from a purely bioinorganic chemistry point of view this challenge needs to be tackled by identifying crucial research questions that are at the heart of better understanding the exposure–response relationship of individual TMs. While only rudimentary silhouettes of these biomolecular mechanisms are currently emerging [4], their gradual completion will provide an important conceptual framework based on which tighter regulations of the emission of TMs into the environment may then be implemented. Reaching this overarching goal will not only allow to better protect babies, children and pregnant women from non-communicable environmental diseases which are caused by TMs that increasingly infiltrate our food supply [8], but also to safeguard industrial workers who are occupationally exposed to TMs during the manufacturing of high tech products [14] as well as dental personnel [15,16].

## 2. Assessment of Human Exposure to TMs

**Figure 1:**
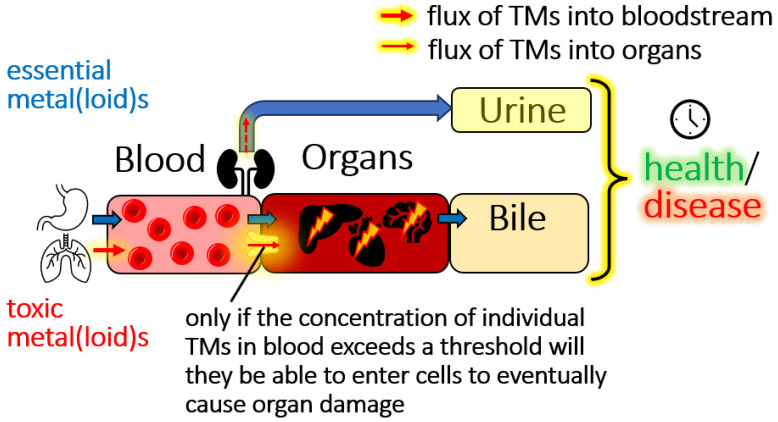
Conceptual depiction of the flow of essential elements (blue arrows) and TMs (red arrows) through a mammalian organism. Crucially, only if a TMs concentration in the bloodstream exceeds a certain threshold will the latter be able to invade target-organ cells to eventually cause organ damage. Since there are 79 organs, the actual target organs of any given TM will be determined by the exposure dose, the threshold concentration of a TMs in the bloodstream, the nutritional status and sex of the individual, the availability of a biomolecular uptake mechanism for TMs metabolites at the target organ surface as well as the density of all uptake mechanisms per mm^2^ at the bloodstream–organ interface. We illustrate mechanisms pertaining to threshold-toxicity, but want to point out that non-threshold toxicity mechanisms also play an important role.

At present, the only means by which environmental and occupational exposure of humans is being addressed is by accurately measuring their blood concentrations [4], which involves agencies, such as the Centers of Disease Control and Prevention (CDC) in the United States and Health Canada. This principle approach allows to unequivocally identify environmental pollutants that have infiltrated human tissues which has matured over the past two decades and is also being referred to as ‘exposomics’ [17,18]. The inherent problem of this approach, however, is the fact that we do not know what the blood concentrations of TMs actually mean in terms of their public health relevance [4]. From a toxicological chemistry point of view one needs to answer a conceptually rather simple, but crucial question, namely what TMs concentrations in the bloodstream of babies, children, pregnant women and industrial workers are tolerable (i.e., no immediate action is needed to change environmental regulations) and which TMs concentrations are concerning for each of these vulnerable populations (i.e., they will trigger the onset of adverse health effects, for example, by inducing deregulated nutrient sensing [19]) (Figure 1). These populations are particularly vulnerable to TMs because (a) babies and children cannot effectively excrete these after the oral ingestion of TM-laced food, (b) the ingestion of TM-laced food by the mother can result in their translocation across the placental barrier to adversely affect the development of the fetal brain and (c) industrial workers are exposed to TMs predominantly via inhalation during their entire time they spend at their workplace of 8 h per day. The biochemical complexity of the bloodstream–organ system in which these dynamic bioinorganic chemistry processes unfold, however, requires the application of powerful research tools to address crucial research questions [20].

### 2.1. Can We Distinguish Between Tolerable and Threshold Toxicity Blood Concentrations of TMs?

Conceptually, every mammalian organism can be regarded as a highly dynamic ‘flow-through cell’ that comprises ~200 cell types through which chemical matter continually flows in form of food and drinking water (i.e., the diet). The latter contain essential trace (Cu, Fe, Zn) and ultra-trace elements (Co, Se, Mo), inherently toxic elements (Cd, Hg, Pb) [2], carbohydrates, fats, proteins as well as vitamins to maintain an individual’s long-term health and wellbeing [21]. Environmental and/or occupational TMs exposure will thus inevitably result in the latter entering the bloodstream to various degrees depending on their molecular form and the degree of their absorption by available uptake mechanisms that are embedded into the enterocytes which line the GI tract (i.e., their bioavailability) [22]. Arguably the most important toxicological chemistry-related question, however, is their organ availability, defined as all biomolecular mechanisms by which TMs are uptaken into target organs as the corresponding total influx in μg TMs/day will ultimately determine if and therefore also when adverse organ-based effects will unfold under chronic exposure conditions.

**Figure 2:**
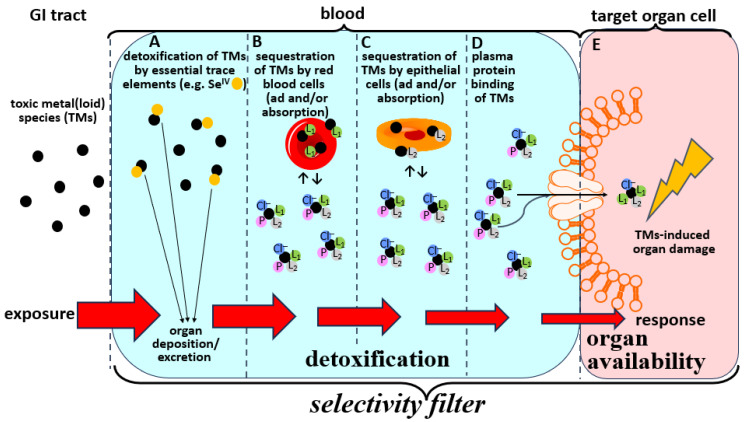
Deliberately simplified conceptual depiction of the biochemical fate of individual TMs in the bloodstream which collectively defines their corresponding exposure-response relationship. Bioinorganic detoxification reactions of TMs in the bloodstream are highlighted in blue, while the uptake of organ available TMs complexes into toxicological target organs is highlighted in pink. Detoxification reactions involve interactions of TMs with essential elements (**A**), their ad/absorption to/into red blood cells (**B**), their ad/absorption by/into endothelial cells (**C**), and their binding to plasma (transport) proteins (**D**). Any residual TMs will interact with ligands in blood plasma such as plasma proteins (P), small molecular weight ligands (L_1_) and chloride ions (Cl^−^) to form organ available complexes that are then translocated via specific biomolecular uptake mechanisms into toxicological target organ cells (**E**). The coordination of H_2_O molecules to the TMs is omitted to enhance clarity. Note that all processes (**A**–**E**) combined effectively constitute the selectivity filters which protect internal organs form the influx of TMs. Only if the TMs dose which enters the bloodstream exceeds the detoxification capacity (**A**–**D**) will they be able to enter toxicological target organs (**E**) and cause organ damage.

Since the human body contains 79 organs, addressing the organ availability for just a single TMs intrinsically requires one to integrate their idiosyncratic blood-based bioinorganic chemistry with the processes that collectively determine its influx into its respective individual target organ (Figure 2). It follows from these contemplations that there likely is a critical threshold blood concentration for every TMs that needs to be exceeded before it may enter any given toxicological target organ to eventually result in the onset of an environmental disease [4]. To distinguish between tolerable and threshold toxicity TMs concentrations in the bloodstream, two conceptual approaches can be considered: an approach that is based on a model animal-based feeding experiment and a biomolecular mechanism-based approach.

The former approach would require one to expose animals to a TMs in the diet and to simultaneously measure the TM concentration in whole blood and in individual organs over time (weeks to months) to identify the maximal blood concentration at which a TMs does not infiltrate individual target organs. To the best of our knowledge such experiments have not been executed, likely because of logistical reasons. While this principle approach should allow one to define a safe TM concentration in the bloodstream (and therefore ipso facto also in the diet), it is practically impossible to extrapolate these results from the chosen animal model to humans.

The latter approach would require one to determine all bioinorganic processes that any given TMs can undergo in the bloodstream-organ system. Conceptually, this includes (a) all detoxification mechanisms in the bloodstream, (b) all blood-based transport mechanisms that translocate an individual TMs to the toxicological target organ surface, and (c) all biochemical translocation mechanisms which orchestrate the influx of individual TMs from the target organ cell surface to the interior of the organ cell (thus the surface density of all available uptake pores per surface area will determine the actual uptake flux of a TMs into any given target organ μg/day). Since it is practically impossible to measure or even estimate these critical parameters for all 79 organs in humans, it is rather unsurprising why we lack the capability to distinguish between tolerable and threshold toxicity concentrations of TMs in the bloodstream. Confronted with this dilemma, the question arises as to what can be done to overcome this fundamental bottleneck?

### 2.2. Bioinorganic Processes of TMs in the Blood-Organ Interface Represent Selectivity Filters

After the infiltration of the bloodstream with TMs, the latter may interact with thousands of possible biomolecular ligands and cells [white blood cells, red blood cells (RBCs)] which thus makes it rather unlikely for the entire absorbed dose to reach any given toxicological target organ(s). In fact, four principle bioinorganic chemistry processes of TMs will unfold in the bloodstream, which may significantly reduce their influx into toxicological target organs. We note that they all unfold simultaneously so the order in which we discuss them is arbitrary.

The first process is the detoxification of the TMs by essential elements (Figure 2A), such as simultaneously ingested dietary selenium species [21]. To this end, it is known that selenite (Se^IV^)—which is present in certain food items [23,24]—can mediate the formation of complexes with Hg-Se and As-Se bonds in the bloodstream [21]. The former complex actually refers to a (HgSe)_100_SelP species that is rapidly formed in blood which may then be deposited in organs and therefore does not pose a toxicological threat [21]. The latter complex refers to the (GS)_2_AsSe^−^ species that is rapidly formed in RBCs [21] and in hepatocytes in vivo [25], which is then rapidly excreted via the bile [21,25,26] and therefore does not pose a toxicological threat to the organism either.

The second bioinorganic process that is relevant is the adsorption of TMs to RBCs [27,28] and/or their sequestration within them [21] (Figure 2B). We will not consider that the concentration of the TMs within RBCs will trigger their rupture as this can—if it happens—result in secondary adverse effects in organs that are beyond the primary scope of this manuscript [29].

The third bioinorganic process of TMs that is conceptually related to their interaction with RBCs, is their ad/absorption by/into endothelial cells (Figure 2C). Since the latter cells cover the interior of all blood vessels, the endothelium represents a considerable surface area that may by itself be regarded as an important toxicological ‘target organ’ for TMs [30,31].

The fourth bioinorganic process that represents a detoxification of TMs in the bloodstream is their binding to plasma transport proteins (Figure 2D). While these interactions were at first—and perhaps naively—thought to solely mediate their translocation to toxicological target organs [32], it is now becoming clear that the actual translocation process is much more complex [33].

The last and arguably most important bioinorganic process of TMs in the bloodstream with regard to better understanding the exposure–response relationship is the interaction of complexes that are formed between TMs and transport proteins and other biomolecules in blood plasma with highly specific biomolecular uptake mechanisms that are present at the surface of 79 organs (Figure 2E) [34]. In principle and to a first approximation the formation of these organ available TM-complexes involves the binding of various ligand(s)(L_x_)—such as a plasma proteins and other biomolecules in plasma—to the TMs of interest, which plays a crucial role in the context of understanding how organ-specific selectivity filters operate at a biomolecular level. Thus, the actual blood-based bioinorganic processes that represent the selectivity filters which are fundamentally involved in mediating the exposure–response relationship (Figure 2A–E) are considerably more complex than some rather simplistic representations that can be found in the literature. To this end, the depiction of the binding of a single ligand (L) to the TMs to form a TM-L complex which is then uptake into the target organ [35] does not describe the complexity of the involved biomolecular processes in sufficient detail (Figure 2A–E).

Taken together, the selectivity filters of TMs in the bloodstream–organ system conceptually entail only three basic processes, namely (a) the bioinorganic chemistry-mediated detoxification of TMs in the bloodstream (Figure 2A–D), (b) the complex coordination chemistry of TMs interacting with multiple ligands in plasma [i.e., plasma proteins, Cl^−^, HCO_3_^−^ and small molecular weight (SMW) species] (Figure 2E) and finally (c) the translocation of the organ available TM-complex that is formed in the blood plasma into the target organ(s), which will ultimately result in the eventual onset of organ damage (‘the dose makes the poison’). Thus, considerable research efforts needed to be directed at addressing the knowledge gaps that pertain to further our understanding of these critical bioinorganic chemistry processes that unfold after a TMs has invaded the bloodstream (Figure 2A–E).

### 2.3. Structural Characterization of Organ Available TMs Species in Blood Plasma

Although the formation of *organ available* TMs species in blood plasma are of the utmost toxicological significance as they ultimately determine all organ-based adverse effects, surprisingly little attention has been directed at structurally characterizing these species. Any effort to structurally characterize these species should start by identifying all ligands that are present in plasma and have an affinity to coordinate to the TM center of interest at the near physiological conditions that are prevalent in blood plasma. Since plasma is an aqueous fluid which contains electrolytes (i.e., ions) as well as biomolecules (i.e., thousands of plasma proteins and ~450 SMW metabolites) one may hypothesize that any organ available TM complex comprises the general formula (L_1_)(L_2_)TMs(L_3_)(L_4_) complex, where all ligands directly coordinate to the metal center. Ligands L_1_ and L_2_ may represent ‘organic’ biomolecules including plasma proteins (PP) as well as small molecular weight (SMW) compounds that are present in human plasma, including amino acids (Cys), small peptides (GSH) and metabolites (hCys) [36]. The blood plasma concentration of Cys and hCys in the blood plasma of healthy adults is in the range of 10–20 μM [33,37,38]. On the other hand, L_3_ and L_4_ may represent ‘inorganic’ ligands including Cl^−^ (~105 mM in plasma), HCO_3_^−^ (~25 mM in plasma) as well as H_2_O molecules (Figure 2E). Although organ available (L_1_)(L_2_)TMs(L_3_)(L_4_) complexes in plasma will be recognized by known uptake mechanisms, such as the divalent metal transporter-1 (DMT1) and ion channels [39], their structural characterization has not received the attention it should [32]. The fact that Cd^2+^ transport into cells, for example, is mediated by transporters that also transport Zn^2+^ and Mn^2+^ suggests that the coordination of a similar set of ligands to these metal ions may explain their cell uptake by a T-type Ca^2+^ channel Cacnα_1G_ [39].

### 2.4. Involvement of SMW Species in Plasma in Mediating the Organ Availability of TMs

Direct experimental evidence in support of an involvement of intravenously administered small molecular weight (SMW) species to animals in the delivery of MeHg^+^ to toxicological target organs has been reported as early as 1982 [40]. With regard to other TMs, such as Hg^2+^, Cd^2+^, Ni^2+^, and Mn^2+^, the coordination of H_2_O molecules as well as Cl^−^ and HCO_3_^−^ ions to the metal center likely play an important role in the formation of organ available TMs in plasma. To this end, it is well known that Hg^2+^ as well as Cd^2+^ react with Cl^−^ form the tetrachloro-complexes, [HgCl_4_]^2−^ as well as [CdCl_4_]^2−^, respectively [41,42]. In this context a LC-based study based on anion-exchange chromatography recently demonstrated that the competitive coordination of Cys to Cd^2+^ in the presence of a 100 mM Cl^−^ containing mobile phase may play a role in the uptake of the latter into toxicological target organs [43]. While the concentrations of Cl^−^ and HCO_3_^−^ ions in plasma will determine to what extent they are coordinated to TMs, the concentrations of plasma proteins (PPs) are known to vary to some degree [44], while the concentrations of certain SMW species, such as hCys in blood plasma vary considerably between healthy individuals (10–20 μM) and hyperhomocysteinuria patients (up to 200 μM hCys) [33,45]. Accordingly, it is possible that the organ availability of certain TMs toward their respective target organs will differ significantly between healthy human adults (hCys plasma concentration: ~10 μM) and hyperhomocysteinuria individuals (hCys plasma concentration: up to 500 μM) as hCys may displace Cl^−^ ligands from the TM center, which will change the stoichiometry of the prevalent (PP)(SMW)TMs(Cl)(H_2_O) complex, which may in turn profoundly change its organ availability [19]. It is therefore possible that the metabolism of TMs which includes their disposition to target organs may differ significantly in hyperhomocysteinemia patients [43].

A critical role of hCys and Cys in the translocation of TMs from blood plasma to target organs has been recently observed when 50 μM concentrations of these SMW metabolites were shown to completely mobilize MeHg^+^ from its binding sites on serum albumin in rabbit plasma to form a MeHg-hCys complex at near physiological conditions [46]. These results strongly suggest that variations in the blood plasma concentrations of these endogenous thiols in individuals may significantly affect the onset of neurotoxic effects following the ingestion of tuna which contains MeHg^+^ [47]. Cys has been recently shown to also be implicated in the translocation of Hg^2+^ and Cd^2+^ from HSA to target organs [33]. The utilization of a metallomics tool allowed to observe a Cys-mediated comparative mobilization of these metals from human serum albumin (has) at near physiological conditions. The results revealed that Hg^2+^ is mobilized from HSA at 50 μM Cys completely, while Cd^2+^ was only partially mobilized. Since these results were obtained using PBS-buffer as the mobile phase they suggest the former TM to be potentially more organ available and therefore also more dangerous from a toxicology point of view.

With regard to the aforementioned results it is also important to recognize that Cys is an amino acid, whose concentration in blood plasma appears to be tightly regulated at 10 μM. In contrast, hCys is actually a metabolite that is the result of the trans-sulfuration pathway which operates in the liver and other organs [48] and whose concentration in blood plasma can be greatly elevated in certain disease processes, such as hyperhomocysteinemia (HHC) [43]. In fact, hCys concentrations in blood plasma of up to 500 μM [49] are not only associated with severe adverse human health effects, such as atherosclerosis and heart disease [45,50,51], but are possibly also associated with an altered metabolism of TMs in terms of their translocation to target organs (e.g., in HHC patients). Furthermore, it must also be considered that the uptake of TMs into target organs may result in the perturbation of the metabolism of sulfur and thus, in turn, adversely affect the metabolism and therefore the concentration of hCys in blood plasma [48].

Taken together, a better understanding of the role that SMW species in plasma impart on the formation of organ available (PP)(SMW)TMs(Cl)(H_2_O) complexes therein is of critical importance to better understand the dose–response relationship of TMs. This research need will require the structural characterization of the (PP)(SMW)TMs(Cl)(H_2_O) complexes that are formed at near physiological conditions of blood plasma [20,32,46,52].

## 3. Modulation of the Bioinorganic Chemistry of TMs in Blood to Mitigate Toxic Effects

**Figure 3:**
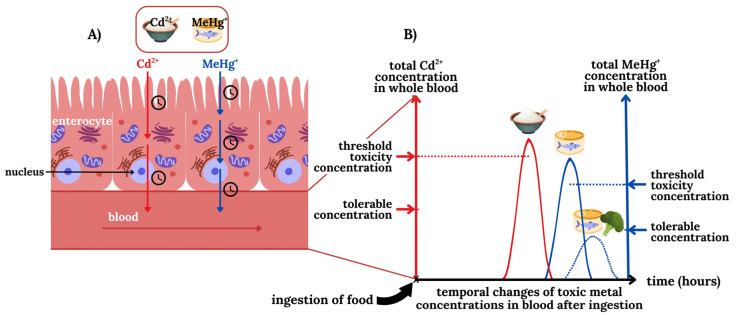
Conceptual depiction of the transport of the TMs Cd^2+^ (red) and MeHg^+^ (blue) from the lumen via enterocytes into the bloodstream (**A**) and the temporal influx of these TMs into the bloodstream (**B**). With regard to the ingestion of food that is contaminated with Cd^2+^ (rice) and MeHg^+^ (tuna) it is important to recognize that owing to their different transport mechanisms the corresponding peak TM blood concentrations will be reached at different timepoints (**B**). Note that only if the blood concentration of Cd^2+^ and MeHg^+^ exceeds their corresponding threshold toxicity concentration will they influx into toxicological target organs and cause damage. The ingestion of TM-laced food items along with foods that contain certain nutrients (e.g., selenium) which affect their organ availability, may be exploited to result in a lower maximum peak TM concentration in the bloodstream. If the latter is below the tolerable blood concentration, adverse organ-based effects may therefore be ameliorated by the ingestion of such a nutraceutical. We note that it is also possible that the tolerable and the threshold toxicity concentration of any particular TMs may be identical.

All of the bioinorganic processes of TMs that unfold in the bloodstream (Figure 2A–E) will collectively determine when the blood concentration of any given gastrointestinally absorbed TMs will eventually reach a maximum concentration therein (Figure 3). Based on concepts that were discussed earlier, the maximum concentration of toxic metal-1 (TM-1) in the bloodstream can either be tolerable (i.e., no influx into target organs will ensue) or it can reach/exceed a threshold toxicity concentration (i.e., its influx into target organs will cause organ damage). It is also likely that a simultaneously ingested but chemically different toxic metal-2 (TM-2) will likely reach its maximal concentrations in the bloodstream at a different time than TM-1 as the kinetics of their uptake across enterocytes are likely to be different. In this context, it is also important to recognize that the ‘peak width’ of the TMs influx into the bloodstream will largely determine if tolerable or threshold toxicity TMs blood concentrations are reached. Accordingly, the co-ingestion of a TMs-contaminated food items with nutrients that are contained in certain foods (e.g., selenium) [53] may significantly affect the bioinorganic processes that are outlined in Figure 2A–E and thus the ‘peak width’ to possibly decrease the maximal TMs concentration that is reached in the bloodstream. If the maximal TMs concentration in the bloodstream would remain below the tolerable blood concentration range their translocation to toxicological target organs may be curtailed, therefore significantly delaying or mitigating the onset of adverse health effects in humans.

## 4. Conclusions

The uncertainty that is associated with the difficulty in causally linking the chronic exposure of humans to TMs with the etiology of grievous environment-related diseases is directly related to our inability to distinguish between tolerable TMs concentrations in blood from threshold toxicity TM concentrations in blood that are likely to trigger the onset of TMs-based environmental diseases. This undesirable situation requires a much better understanding of the corresponding selectivity filters which protect internal organs from the influx of individual TMs and thus play a fundamental role in determining the onset of organ damage. To highlight the importance of how these selectivity filters for TMs operate, we have deconstructed them into their essential components, namely the bioinorganic detoxification processes that unfold in the bloodstream and those which mediate the uptake of organ available TMs complexes into target organs. Based on this integrative view, three critical research questions are identified that need to be better understood. The first refers to bioinorganic detoxification processes of TMs that unfold in whole blood and involve essential elements (e.g., their detoxification by selenium compounds and/or other essential elements). The second refers to the need to better understand the sequestration of TMs by RBCs and/or endothelial cells. Last but not least the TMs metabolites that are assembled in the bloodstream and represent the substrates that are uptaken into target organs need to be structurally characterized. Advancing our understanding of each of these bioinorganic processes that collectively constitute the corresponding selectivity filters will contribute to better understand their exposure-response relationships, which is seen by many as a fundamental challenge in the post genomic world we live in [54,55] and represents an important effort to more effectively address the mixture toxicity problem [56,57], which refers to the fact that all human populations are simultaneously exposed to multiple TMs. Progress toward addressing these challenges will serve as the starting point to recommend strategic approaches to integrate chemical safety into health systems to reduce the disease burden and improve public health outcomes particularly in low and middle income countries and worldwide [58,59,60,61,62].

## Data Availability

No new data were created or analyzed in this study.
